# Immunoregulatory, proliferative and anti-oxidant effects of nanocurcuminoids on adipose-derived mesenchymal stem cells

**DOI:** 10.17179/excli2019-1366

**Published:** 2019-06-17

**Authors:** Forouzan Yousefi, Fahimeh Lavi Arab, Mahmoud Reza Jaafari, Maryam Rastin, Nafiseh Tabasi, Mahdi Hatamipour, Karim Nikkhah, Mahmoud Mahmoudi

**Affiliations:** 1 Immunology Research Center, Mashhad University of Medical Sciences, Mashhad, Iran; 2 Biotechnology Research Center, Pharmaceutical Technology Institute, Mashhad University of Medical Sciences, Mashhad, Iran; 3 Department of Pharmaceutical Nanotechnology, School of Pharmacy, Mashhad University of Medical Sciences, Mashhad, Iran; 4 Immunology Research Center, BuAli Research Institute, Faculty of Medicine, Mashhad University of Medical Sciences, Mashhad, Iran; 5 Nanotechnology Research Center, Mashhad University of Medical Sciences, Mashhad, Iran; 6 Department of Neurology, Faculty of Medicine, Mashhad University of Medical Sciences, Mashhad, Iran; 7 Department of Immunology, Faculty of Medicine, Mashhad University of Medical Sciences, Mashhad, Iran

**Keywords:** immunoregulatory, antioxidant, nanocurcuminoids, adipose-derived mesenchymal stem cells

## Abstract

Curcuminoids are dietary complexes extracted from the seeds of *Curcuma longa L*. that contain curcumin, bisdemethoxycurcumin and desmethoxycurcumin. Curcuminoids are popular for their pleiotropic therapeutic functions, such as their anti-inflammatory and anti-oxidant effects. Nonetheless, their clinical use is associated with poor systemic bioavailability and insolubility. The nano-formulation of curcuminoids eliminates these shortcomings. In the present study, we explored immunoregulatory, proliferative and anti-oxidant effects of nanocurcuminoids on adipose-derived mesenchymal stem cells (AT-MSCs). Flow cytometry analysis and MTT assay were employed to explore the effects of nanocurcuminoids on the apoptosis and proliferation of adipose-derived MSCs (AT-MSCs). The anti-oxidant effect of nanocurcuminoids on AT-MSCs also was examined. The immune regulatory effect of nanocurcuminoids was evaluated by the flow cytometric measurement of the T regulatory (Treg) population. The expression of inflammatory and anti-inflammatory cytokines was quantified using real-time PCR. Our findings demonstrate that low concentrations of nanocurcuminoids are beneficial for MSC proliferation, protection of MSCs from apoptosis, reducing inflammatory cytokines and SOD activity. A high concentration of nanocurcuminoids increases the population of Tregs and elevates the expression of TGFβ and FOXP3 genes. The beneficial effects of nanocurcuminoids on AT-MSCs were mainly observed at low doses of nanocurcuminoids.

## Introduction

Mesenchymal stem cells (MSCs) are multipotent self-renewing cells that are able to differentiate toward various cell/tissue lineages. MSCs generate a growth-enhancing secretome with anti-inflammatory and antioxidant agents, because of which they are considered as an appropriate candidate for use in regenerative medicine (Carbone et al., 2016[5]; Ge et al., 2015[12]). 

The use of natural herbs to remedy disease has attracted considerable attention over the past decade. Among the natural pharmacological products, curcumin has been shown to exhibit anti-oxidant, immunomodulatory and anti-tumor activity and is considered to be a potential modality for treatment of various inflammatory and neurodegenerative diseases (Fadus et al., 2017[11]; Ghasemi et al., 2016[13]; Moradzadeh et al., 2018[29]).

Curcuminoids are natural polyphenols extracted from the dried rhizomes of *Curcuma longa L.* (turmeric) that consist of curcumin, bisdemethoxycurcumin and desmethoxycurcumin, which are together known as C3 complex. The main component of curcuminoids, curcumin has long been used in Middle Eastern traditional medicine. Because of its low molecular weight, curcumin is capable of crossing the blood-brain barrier and it plays a defensive role against oxidative stress observed in neurodegenerative disease through the scavenging of oxidizing materials (Huang et al., 2018[16]; Mirzaei et al., 2017[27]).

Despite its pharmacological properties, the clinical use of curcumin is limited by factors such as poor bioavailability, low aqueous solubility and rapid clearance from the body, making its therapeutic application problematic (Liu et al., 2016[23]). Delivery systems based on nanoparticles, such as liposomes or micelles, can solve these problems and increase the bioavailability of curcumin (Chidambaram and Krishnasamy, 2014[7]; Pandit et al., 2015[33]; Silva et al., 2018[41]). 

In this scenario, micellar-based carriers of relatively small size with an amphiphilic construction and core-shell shape, offer several advantages as drug delivery systems for transmitting lipophilic therapeutic agents. Micelles can eliminate the key limitations of curcumin, such as its unstable structure, insolubility and rapid metabolism. Surprisingly, curcuminoids can remain in nano-micelle compounds for a long time, enabling them to exert their beneficial effects before rapid intestinal digestion. A variety of copolymers can be inserted into micelles to develop nanocarrier-based micelles for therapeutic purposes (Li et al., 2018[22]; Schiborr et al., 2014[40]). 

In our recently published work, the safety and efficacy of nanomicelle curcuminoids on subjects with ALS and diabetes was demonstrated (Ahmadi et al., 2018[1]; Rahimi et al., 2016[37]). It also was shown that oral absorption of this formulation of nanocurcuminoids by mice is at least 50 times greater than that of the conventional curcumin powder. Nanomicelles increased the solubility of curcuminoids more than 100,000 fold (International application No. PCT/IB2018/051370; International filing date: 4 March 2018).

In the present study, the efficacy of nanocurcuminoids was explored on the cell proliferation and apoptosis of AT-MSCs. Furthermore, we investigated whether the micelle-encapsulated curcuminoids could increase proliferative, anti-apoptotic, anti-oxidant and immunomodulatory effects in adipose-derived mesenchymal stem cells (AT-MSCs).

## Materials and Method

### Nanocurcuminoids preparation

Curcuminoid nanomicelles were prepared using generally recognized as safe (GRAS) excipients (Ahmadi et al., 2018[1]; Rahimi et al., 2016[37]). The encapsulation efficiency of the curcuminoids in the nanomicelles was almost 100 %. The mean diameter of nanomicelles was approximately 10 nm as determined by dynamic light scattering. The content and size distribution of the nanomicelles in the curcuminoids remained constant for at least 24 months. 

### Human adipose tissue isolation and MSC culture 

Subcutaneous abdominal fat from seven female subjects was isolated using liposuction surgery in a safe and well-tolerated manner. The lipoaspirate tissues were washed with equal volumes of phosphate-buffered saline (PBS; Gibco) and the suspended cells were submitted to enzymatic digestion with collagenase I (1 mg/mL; Worthington; USA) and incubated for 45 min at 37 °C. Afterward, collagenase activity was stopped by adding Dulbecco's Modified Eagle's Medium (DMEM; Atocel; Austria) supplemented with 10 % fetal bovine serum (FBS; PAN Biotech; origin: Australia human grade, gamma radiate) plus 1 % penicillin/streptomycin (100 units/mL)/ (100 μg/mL) (Gibco) into the collected cell suspension. 

The resulting cell solution was then transferred to T175 culture flasks (SPL Life Sciences; Korea) and maintained in a humidified incubator with 95 % air and 5 % CO_2_ incubator at 37 °C. The medium was replenished after day 2 to remove floating debris and the flasks were fed with fresh medium. When the adherent cells became 90 % confluent, the cells were rinsed with PBS and detached using 0.25 % trypsin-EDTA (Gibco). Next, the detached cells were sub-cultured for further cell expansion for the cell therapy of patients with MS in another work and the cells from the second passage were harvested and seeded into six-well plates at a final density of 1×10^4 ^cells/cm^2^ for assay. The study was approved by the Ethics Committee of Mashhad University of Medical Sciences and written informed consent was obtained from all subjects before conducting the experiments.

### Surface marker characterization of AT-MSCs by flow cytometry 

Prior to cell treatment, superficial characterization of AT-MSCs was performed according to manufacturer recommendations. The adherent, spindle-shaped MSCs were harvested using 0.25 % trypsin-EDTA and the trypsin activity was neutralized in complete medium containing FBS. The resultant cells were suspended in PBS containing 5 % FBS. The AT-MSCs were identified by the absence of FITC-conjugated APC-CD45, PE-CD31, FITC-CD14, PE-CD34, FITC-human leukocyte antigen (HLA-DR) and the presence of FITC-CD90, FITC-CD73 and PE-CD105. Isotype-matched antibodies were employed as controls. All antibodies were purchased from eBioscience (USA). Cell aliquots consisting of 8×10^5^ cells/tube were incubated in the dark for 45 min at 4 °C. After washing with PBS, they were acquired on a FACSCalibur flow cytometer (Becton Dickinson). FlowJo software was used to analyze the data. 

### In vitro adipogenic potential of AT-MSCs

In order to confirm the adipogenic potential of AT-MSCs after they had grown to 90 % confluence, they were trypsinized in trypsin/EDTA and centrifuged for 5 min at 1200 rpm. The resulting cell mixture was plated at 5×10^4^ cells/mL on six-well culture plates in the presence of DMEM supplement with 10 % FBS. After 48 h, the medium was replaced with adipogenic differentiation media (AdipoDiff; StemMACS) including DME + 10 % FBS, 2 mM l-glutamine, antibiotic (100 U/mL penicillin, 100 µg/mL streptomycin), 10^7^M dexamethasone, 50 g/mL of ascorbic acid, and 10 g/mL of indomethacin. The plates were maintained in a humidified incubator (37 ºC, 95 % CO_2_) for three weeks. The medium was refreshed every three days. Non-stimulated cells were kept in the DMEM + FBS media alone as the control. After 21 days, the cells were subjected to staining with Oil Red O for microscopic detection of lipid vacuoles.

### In vitro osteogenic potential of AT-MSCs

To detect the osteogenic potential of the AT-MSCs, the cells were cultivated at a density of 5×10^4^ cells/mL in six-well plates after trypsinization. Next, they were incubated in a complete medium consisting of DMEM enriched with 10 % FBS for 48 h. Afterwards, the medium was exchanged with osteogenic induction medium (OsteoDiff; StemMACS) containing 1 nM dexamethasone, 2 mM β-glycerophosphate and 50 µM ascorbate-2-phosphate. The cells then were incubated for approximately 21 days in a humidified incubator (37 °C at 95 % CO_2_). The medium was replaced every three days. Finally, mineralization was observed after application of Alizarin Red (pH = 4.1) and alkaline phosphatase staining according to recommended protocols.

### In vitro chondrogenic potential of AT-MSCs

The chondrogenic potential of the AT-MSCs was established using the pellet culture method with standard protocols. The trypsinized cell aliquots (800,000 cells) were centrifuged twice in a 50 mL centrifuge tube (5 min; 500 g) to generate a cell pellet. After 24 h, the basal medium was gently replaced with chondrogenic induction medium containing 40 μg/mL of proline (Thermo Fisher Scientific; USA), 10 ng of insulin, 10 ng/mL of transforming growth factor-β3 (TGF-β3) (Peprotech; USA), 50 μg/mL of ascorbic acid-2-phosphate, 100 nM of dexamethasone and DMEM with 1 % ITS + Premix (BD Biosciences; USA). The medium was carefully replenished every four days without disturbing the aggregated cells. After 21 days, the cells were fixed with 4 % paraformaldehyde and the extracellular matrix was examined. The chondroitin cryo-sections were evaluated using toluidine blue and hematoxylin-eosin staining to detect glycosaminoglycans (GAGs) and proteoglycans resulting from chondrogenic differentiation.

### Peripheral blood mononuclear cell (PBMCs) preparation

Peripheral venous blood samples of healthy subjects were collected and, after density gradient centrifugation using Ficoll (Lymphodex, inno-train), the surface layer was removed and the buffy coats were separated and washed twice in PBS containing 5 % FBS. The resulting PBMCs were used for subsequent testing. Trypan blue was used for microscopic determination of viable PMBCs.

### Treatment of AT-MSCs 

Harvested MSCs derived from abdominal fat were plated onto six-well plates containing 2 mL of DMEM + 10 % FBS at a final density of 1×10^4 ^cells/cm^2^. Approximately 60 h after cell seeding, when the AT-MSCs reached 60 % confluence, nanocurcuminoids were added in different concentrations (0.5, 1, 2.5, 5, 12, 25, 50, and 100 µM) to their corresponding wells and incubated for 24 h in a humidified incubator (37 °C, 95 % CO_2_). Control samples were divided into three groups: AT-MSCs + micelles only, AT-MSCs + free curcumin, and un-treated AT-MSCs. 

### Mixed lymphocyte culture (MLR)

To identify the synergistic effect of AT-MSCs and nanocurcuminoids on the enhancement of TCD4+CD25+FOXP3+CD127-cells (Tregs), when MSCs reached 60 % confluence, PBMCs were added to AT-MSCs pretreated with different doses of nanocurcuminoids at a 1:5 ratio (MSCs:PBMCs). In this co-culturing system, the stimulator cells were AT-MSCs and the PBMCs were the responders. DMEM supplemented with 10 % FBS was used to feed the cells. 

### Cell proliferation assay by MTT 

AT-MSCs (1×10^5^ per well) were seeded in six-well flat-bottom plates (Iwaki SciTech; Japan) and allowed to achieve 50 % to 60 % confluence. The medium was replaced with 2 mL of complete culture medium (DMEM + FBS) a different concentrations (0.5, 1, 2.5, 5, 12, 25, 50, and 100 µM) of nanocurcuminoids were inserted into each well. Twenty-four hours after nanocurcuminoid incubation, cell proliferation was measured by MTT ((4, 5-dimethylthiazol-2-yl)-2, 5-diphenyltetrazolium bromide) assay. Briefly, 30 µL of MTT solution (5 mg/mL) was added to each well and the plates were maintained in a CO_2_ incubator for 5 h. The medium was gently removed and 200 µL of DMSO was added to the wells. Absorbance was read using an ELISA reader at 570 nm. 

### Apoptosis assay using annexin V/propidium iodide (PI) dual staining

The modulatory effect of nanocurcuminoids on the rate of MSC apoptosis was quantitatively examined by flow cytometry using an Annexin V-FITC Apoptosis Detection Kit (Molecular Probes; Germany). Briefly, 24 h after the treatment of AT-MSCs with different doses of nanocurcuminoids, all plated cell groups, including the samples and controls, were harvested using trypsin-EDTA solution, washed and resuspended in 300 μL of binding buffer. Resultant cells were incubated with 5 μL of annexin V-FITC for 30 min at 37 °C in the dark. Subsequently, the cells were incubated with 5 μL of PI for 15 min. Afterwards, the cells in early and late apoptosis stages were distinguished using flow cytometric dot plot graphs. Early apoptotic cells were determined as annexin+/PI, late apoptosis as annexin+/PI+ and necrotic cells as annexin-/PI+.

### Measurement of TCD4+CD25+FOXP3+ CD127^-^ cells by flow cytometry

To investigate the synergistic effects of the nanocurcuminoids and AT-MSCs on the expansion of CD4+CD25+FOXP3+CD127**^-^** T cells, after co-culturing PBMCs and AT-MSCs for 24 h in the presence of serial concentrations of nanocurcuminoids, the PBMCs from all concentrations were collected and immunolabeled with monoclonal antibodies against FITC-CD4, PE-CD25, PEcy5- FOXP3, APC-CD127 and an isotype-matched control according to manufacturer protocol (eBioscience; USA). Next, the cells were initially gated by a forward/side scatter and set with the isotypic control. About 100,000 cells/tube were acquired on a FACSCalibur flow cytometer (BD Biosciences) and analyzed in FlowJo.

### RNA isolation and quantitative real-time PCR analysis

Real-time PCR was performed to quantify mRNA expression of inflammatory and anti-inflammatory cytokines. For this purpose, after 24 h of treatment with different doses of nanocurcuminoids, the total RNA from the mixture of PBMCs + MSCs was extracted using TRIzol reagents (Roche Diagnostics; Germany) and complementary DNA was synthesized according to manufacturer protocol using a PrimeScript RT kit (Takara Biotechnology; Japan). Each cDNA was amplified using a SYBER Green PCR mix (Wizpure qPCR Master; Korea) in a Rotor-Gene 6000 thermal cycler (Qiagen; Germany) system. The mean cycle threshold (CT) of the samples was measured with normalization to glyceraldehyde-3-phosphate dehydrogenize level as the internal control reference gene. All data are expressed as mean fold change compared to un-treated cells (control), calculated using the 2^-∆∆Ct^ formula. ∆∆Ct was determined based on differences in the CT from the targeted and internal genes. The cut-off values for appraising the fold changes of gene expression were estimated according to CT from the control group.

### Assessment of NO production

After seeding the AT-MSCs (1×10^5^ cells/well) in six-well plates and allowing them to reach 50 % to 60 % confluence, various nanocurcuminoid doses (0.5-100 µM) were added to the wells. At 24 h post-treatment, the culture supernatants were collected to evaluate the effect of nanocurcuminoids on inhibiting NO production. For this purpose, 300 µL/tube of the condition medium was centrifuged (5000 rpm) based on manufacturer protocol (ZellBio; Germany). The NO concentration was evaluated using a microplate/ELISA reader at 550 nm. The OD value of the unknown samples was placed in the linear regression equation to calculate the final NO value (µM).

### Measurement of malondialdehyde (MDA)

To determine the inhibitory effect of nanocurcuminoids on lipid peroxidation, after the pretreatment of AT-MSCs with different doses of nanocurcuminoids (0.5, 1, 2.5, 5, 12, 25, 50, and 100 µM) for 24 h, 50 µL/sample of the supernatant from each well was isolated and centrifuged (3500 rpm) according to manufacturer protocol of the kit (ZellBio; Germany). The MDA level (µM) was identified by matching OD from unknown samples with equivalent numbers on the standard curve. OD was read at 535 nm using the ELISA reader. 

### Measurement of superoxide dismutase (SOD)

To evaluate the antioxidant activity of nanocurcuminoids in terms of promoting SOD activity, 10 µL of the condition medium was taken from all concentrations and control groups. Afterwards, 10 µL of chromogen along with the other reagents was added to each well according to the kit manual (ZellBio; Germany). In the blank specimen, all reagents were added except for chromogen. Each sample was utilized as its blank. The chromogenic end product was acquired on the microplate/ELISA reader at 420 nm at 0 min and 2 min. The SOD activity was calculated according to the following formula:

SOD activity (U/ml) = V_p_-V_c_/V_p_×60 

V_p_ = OD_ sample 2min_-OD_ blank 2min_

V_c_ = OD_ sample 0 min_-OD _blank 0min_

### Statistical analysis

The data was reported as mean ± standard deviation (SD) and the comparison between values was performed by one-way analysis of variance (ANOVA) in GraphPad Prism 6 software. A value of p < 0.05 was accepted as significant (*) and p ≤ 0.01 was considered as highly significant (**). The reported comparison is between treated cells and control groups, including untreated cells, micelles only and naïve curcumin groups. Tukey's test was utilized as a post-test for multiple comparisons between the mean values of each group.

## Results

### Isolation and immunophenotyping of AT-MSCs

Lipoaspirate tissue from seven female individuals were successfully obtained at a mean volume of 194.28 ± 16.1 mL. After collagenase digestion and cell harvesting, they reached 70 % confluence within seven days, displaying a homogenous population of fibroblast-like and polygonal morphology with no abnormal change in phenotype. The samples from the culture medium and cells showed no contamination with mycoplasma or aerobic bacteria. Two T175 flasks were subcultured for the assays in the present study. The flow cytometry analysis of AT-MSC surface markers at the first and second passages revealed the co-expression of CD105, CD73 and CD90 and were negative for CD31, CD14, CD34, CD45 and HLA-DR (Supplementary Figure 1).

### Differentiation potential of AT-MSCs

After maintaining the AT-MSCs in the specialized induction medium for three weeks, the trilineage capacity of the AT-MSCs for differentiation into adipocytes, osteocytes and chondrocytes was confirmed (Supplementary Figures 2a-2f). Cytoplasmic lipid droplets as feasible markers of adipogenesis were detected after staining with Oil Red O on day 16 of the culture (Supplementary Figure 2b). The chondrogenic potential of the AT-MSCs was verified using the micromass culture system. Chondroitin cryo-sections were used to observe cartilage-derived GAGs and sulfated proteoglycans resulting from chondroitin fragments using toluidine blue and hematoxylin-eosin staining (Supplementary Figures 2e and 2f). Bone mineralization was determined in differentiated MSCs by the appearance of extracellular calcium deposits in bright orange-red. The other bone formation indicator was detected by the appearance of intense dark-blue-violet staining resulting from alkaline phosphatase activity (Supplementary Figures 2c and 2d). Control cells did not exhibit any feature of differentiation following staining. 

### Nanocurcuminoids at very low concentrations protect AT-MSCs from apoptosis

**Figure 1:**
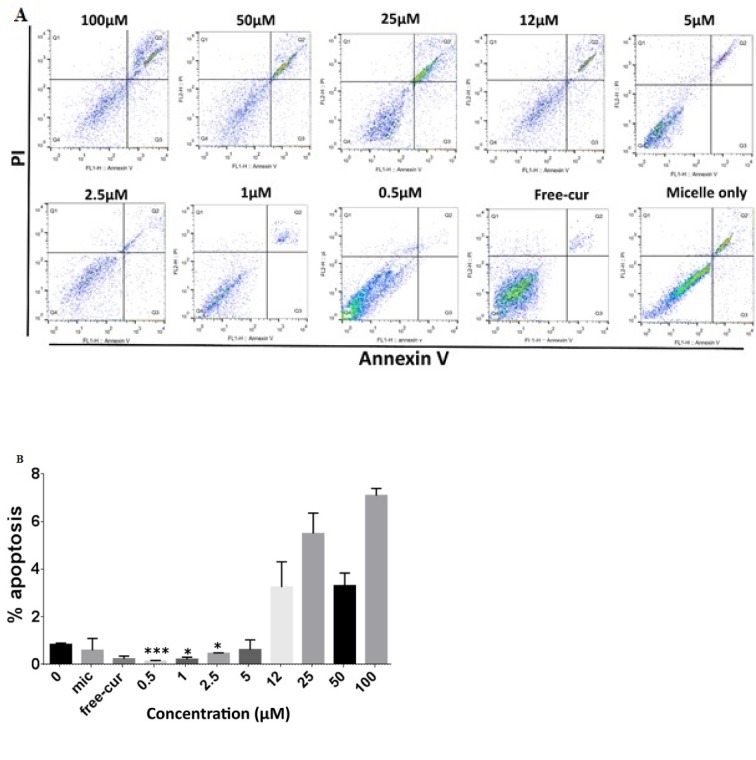
A. Flow cytometry analysis of apoptotic cells that evaluated using Annexin V and propidium iodide double-staining. Representative dot plots showing the percentage of late apoptosis in MSCs after pretreatment with low doses (0.5, 1, and 2.5 µM) of nanocurcuminoids decreased substantially compared to untreated cells and those treated with high doses (12, 25, 50, and 100 µM) (x-axis: green fluorescence of annexin-V-FITC showing apoptotic cells; y- axis: red fluorescence of PI showing necrotic cells). B. Quantitative analysis of apoptosis as shown in A. *significantly different from 0 µM (untreated cells), *p ≤ 0.05, ***p ≤ 0.001. Data shown as mean ± SD from seven independent experiments.

To explore whether or not nanocurcuminoid-driven AT-MSC proliferation was linked with the rate of apoptosis, an apoptosis assay was performed to determine the cell death index of the AT-MSCs. After seeding the AT-MSCs onto six-well plates, the cells were treated with different concentrations (0.5-100 µM) of nanocurcuminoids and the percentage of naturally occurring apoptotic cells was determined after double-staining of death markers (annexin V and PI) using flow cytometry assay. Dot plot flow cytometric graphs show that nanocurcuminoids at low doses (0.5, 1, and 2.5 µM) significantly reduced apoptosis compared with the non-exposed AT-MSCs (p ≤ 0.05; Figure 1(Fig. 1)). Nanocurcuminoids exhibited logical behavior with regard to the apoptosis and proliferation platform of the AT-MSCs. This means that, at low doses, nanocurcuminoids noticeably protected the cells from apoptosis in a dose-dependent manner (p ≤ 0.05) and, at the mentioned concentrations, stimulated AT-MSC proliferation dose-dependently. 

### Low doses of nanocurcuminoids stimulate proliferative capacity of AT-MSCs

**Figure 2:**
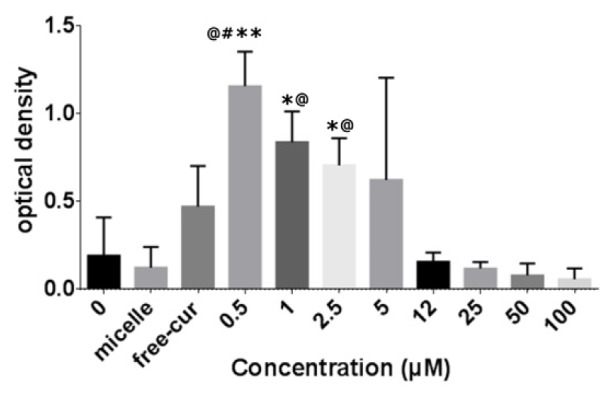
Rate of AT-MSC proliferation after incubation with increasing doses of nanocurcuminoids (0-100 µM). Nanocurcuminoids at low concentrations (0.5, 1, and 2.5 µM) significantly stimulated AT-MSC proliferation when compared with control groups (untreated cells and micelle only-treated cells). *significance vs. untreated cells (p ≤ 0.05), # significance vs. free curcumin (p ≤ 0.05), @ significance vs. blanked micelle (p ≤ 0.05). The data is presented as mean ± SD for seven independent experiments.

The proliferation index of AT-MSCs was evaluated after incubation with increasing concentrations (0.5, 1, 2.5, 5, 12, 25, 50, and 100 µM) of nanocurcuminoids. The results indicate that nanocurcuminoids dose-dependently stimulated MSC proliferation. Nanocurcuminoids at lower doses (0.5, 1, and 2.5 µM) substantially stimulated AT-MSC proliferation (p ≤ 0.01) compared with the control groups (blanked micelle and untreated AT-MSCs) and increased the mean rate of AT-MCC expansion approximately 10-fold at these doses. At higher concentrations (100, 50, 25, and 12 µM), nanocurcuminoids did not change or decrease AT-MSC proliferation. Furthermore, 0.5 µM of nanocurcuminoids augmented cell growth more than did free curcumin. Free curcumin also increased the rate of AT-MSC proliferation compared to non-induced cells, micelles only and high doses of nanocurcuminoids, but this increase was not statistically significant (Figure 2(Fig. 2)). 

### High concentrations of nanocurcuminoids upregulate frequency of TCD4+CD25+ FOXP3+CD127- 

**Figure 3:**
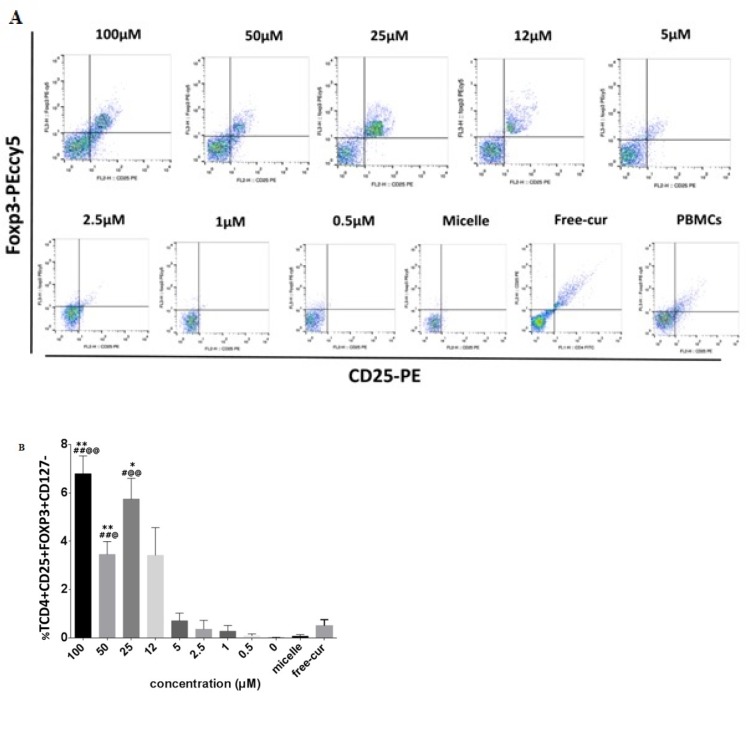
A. Flow cytometry analysis of Treg population that evaluated using extracellular (CD4, CD25 and CD127) and intracellular (FOXP3) markers. Dot plot graphs show that frequency of Tregs after co-culturing of MSC/PBMCs for 24 h increased significantly at higher concentrations of nanocurcuminoids (25, 50, 100 µM) (p ≤ 0.05) compared with lower doses (0.5, 1, 2.5, and 5 µM). B. Quantitative analysis of Treg population as shown in A.*level of significance in treated cells compared to those without nanocurcuminoids (0 µm); # level of significance vs. free-cur; @ significance vs. micelle-only treated cells. Data is shown as mean ± SD from seven independent experiments.

To establish the effect of nanocurcuminoids on the immunoregulatory activity of AT-MSCs, PBMCs were co-cultured with AT-MSCs in the presence of escalating concentrations (0.5-100 µM) of nanocurcuminoids for 24 h. The PBMCs then were isolated for flow cytometric staining of CD4, CD25, CD127 and FOXP3 as known markers of Tregs. Surprisingly, dot plot flow cytometric graphs indicated that elevated doses of nanocurcuminoids (100, 50, and 25 µM) significantly augmented the Treg proportion (p ≤ 0.05) in a dose-dependent manner compared to the control groups (free-cur, empty micelle and untreated cells (MSCs + PBMCs)) with maximum efficacy at 100 µM of nanocurcuminoids and minimum efficacy at 0.5 µM. Concentrations of nanocurcuminoids below 12 µM reduced the Treg percentage dose-dependently (Figure 3(Fig. 3)).

### Anti-inflammatory effect of nanocurcuminoids is authenticated at low concentrations 

To further explore the immunomodulatory effect of nanocurcuminoids on AT-MSCs, the mRNA expression of both inflammatory and anti-inflammatory cytokines was quantitated using real-time PCR. To this end, after 24 h of treatment at different doses of nanocurcuminoids, the total RNA from the PBMC/MSC mixture was extracted and reverse-transcribed to produce complementary DNA as a sample for the amplification of mRNA expression. The results revealed that nanocurcuminoids at low doses (below 12 µM) are better able to decrease the expression of inflammatory cytokines, while at high concentrations (12-100 µM), they elevated the expression of TGFβ and FOXP3. The level of IL17, IFNγ and IL6 expression decreased significantly (p ≤ 0.05) at low doses (0.5, 1, 2.5, and 5 µM) of nanocurcuminoids compared with the controls (untreated cells).

**Figure 4:**
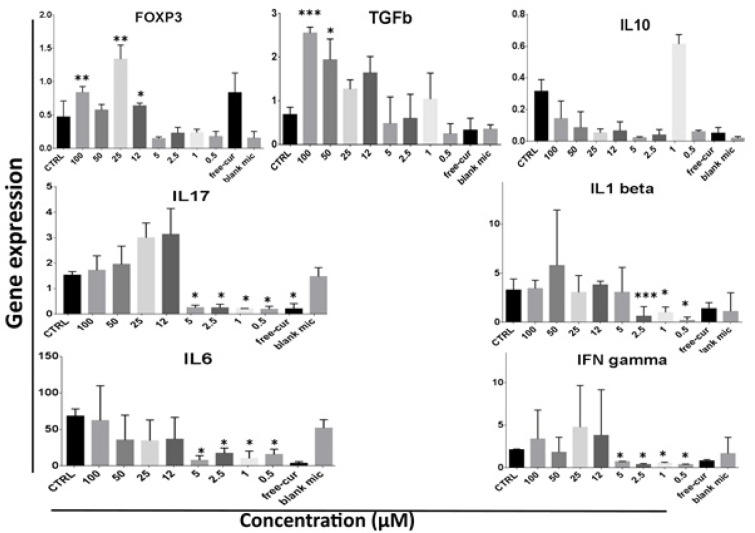
Level of inflammatory (IL17, IL6, IL1b and IFN gamma) and anti-inflammatory cytokine expression (TGFβ and IL10) as well as expression of FOXP3 as the intracellular marker of Tregs. *level of significance of treated cells compared to those without nanocurcuminoids (0 µM). Data is shown as mean ± SD from seven independent experiments.

Nanocurcuminoids also significantly decreased IL17 expression at a level comparable to 0.5 µM. Furthermore, the expression of IL1b was substantially attenuated (p ≤ 0.05) at 1, 2.5 and 5 µM of nanocurcuminoids compared to the control. Naked curcumin also significantly reduced IL1b expression with respect to the control. Conversely, nanocurcuminoids at higher doses (50 and 100 µM) noticeably augmented TGFβ expression (p ≤ 0.05) compared to the control. The level of FOXP3 expression increased significantly at 12, 25 and 100 µM of nanocurcuminoids (p ≤ 0.05). Although naïve curcumin also raised the FOXP3 level, this increase was not statistically significant. Finally, the expression of IL10 was not significantly affected by nanocurcuminoids (Figure 4(Fig. 4)). 

### Protective effect of nanocurcuminoids against membranous lipid peroxidation by reducing MDA level 

**Figure 5:**
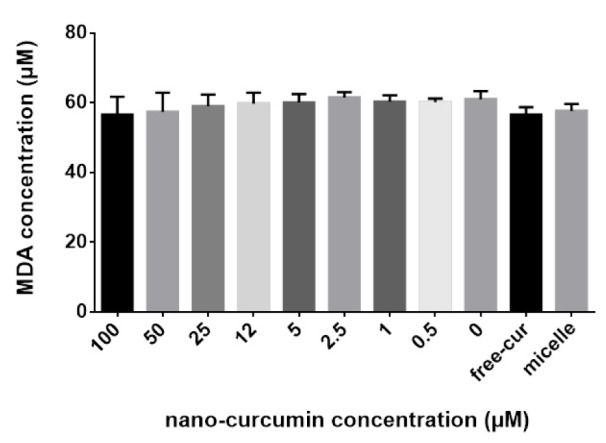
Figure 5: Anti-oxidant effect of nanocurcuminoids for protection against lipoperoxidation resulting from MDA accumulation. No detectable difference between the concentrations of nanocurcuminoids was observed. Data is shown as mean ± SD from seven independent experiments.

One anti-oxidant-mediated effect of nanocurcuminoids is its lipid-peroxidation effect. The extent of lipid peroxidation after exposure to serial concentrations of nanocurcuminoids was quantitatively measured by determining the extracellular MDA level as a final end-product of lipid peroxidation. The spectrophotometric outcomes obtained from the MDA assay did not show a significant change in MDA level at different concentrations of nanocurcuminoids. Nonetheless, doses of 50 and 100 µM of nanocurcuminoids decreased the MDA content of MSC-derived condition medium in a non-significant manner. Free curcumin also non-significantly decreased the lipid peroxidation index of MSCs compared to that of the untreated cells, micelles only and low concentrations of nanocurcuminoids (Figure 5(Fig. 5)).

### SOD activity was augmented at low doses of nanocurcuminoids 

**Figure 6:**
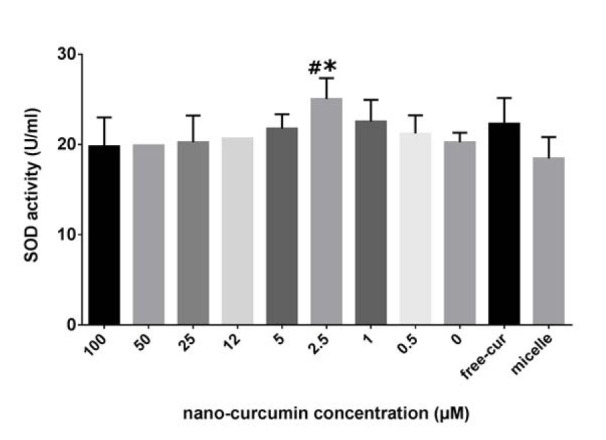
Superoxide dismutase activity of MSC-arisen supernatant after exposure to various doses of nanocurcuminoids showing no significant difference among nanocurcuminoid-stimulated and non-stimulated cells. Only 2.5 µM of nanocurcuminoids significantly elevated SOD activity compared to unstimulated and micelle-only cells (p ≤ 0.05). *level of significance in treated cells compared to those without nanocurcuminoids (0 µM), # significance vs. micelles-only treated cells. Data are shown as mean ± SD from seven independent experiments.

To elucidate the effective dose of nanocurcuminoids for increasing SOD activity as an anti-oxidant enzyme, AT-MSCs were exposed to escalating concentrations (0-100) of nanocurcuminoids for 24 h. The results revealed no measurable difference between the doses of nanocurcuminoids, but there was an increase in SOD activity at 2.5 µM of nanocurcuminoids compared to un-stimulated and micelle-only treated cells (p ≤ 0.05) (Figure 6(Fig. 6)). 

### Efficacy of nanocurcuminoids on NO production 

**Figure 7:**
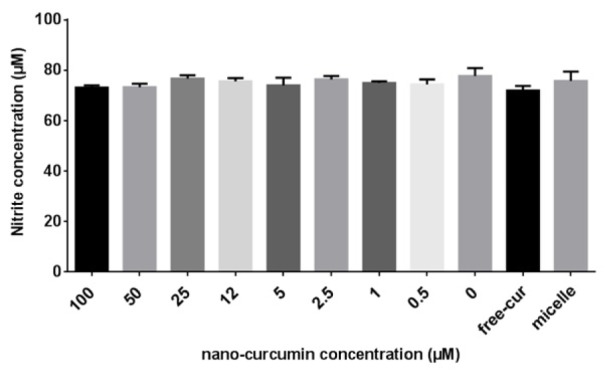
Extracellular nitrite content after exposure to increasing doses of nanocurcuminoids (0-100). No significant difference between concentrations of nanocurcuminoids was found. Data is shown as mean ± SD of seven independent experiments.

To test whether the oxidant scavenging effects of nanocurcuminoids is related to a decrease in NO level and to determine the optimal dose of nanocurcuminoids for this effect, various concentrations of nanocurcuminoids were added to the culture wells. After 24 h, the extracellular NO level was quantitatively measured using a commercially prepared NO kit. No detectable difference was observed between various concentrations of nanocurcuminoids in terms of reducing NO content. Although free curcumin, 50 and 100 µM of nanocurcuminoid-exposed cells lowered NO, this decrease was not statistically significant (Figure 7(Fig. 7)).

## Discussion

Based on their pleiotropic functions, particularly in cancer chemo-preventive applications, curcuminoids have been used extensively in herbal-driven medicine for the treatment of various diseases. Recently, the anti-inflammatory, antioxidant and neural growth-promoting properties of curcumin have attracted considerable attention for the treatment of disease (Qureshi et al., 2018[35]). Accordingly, curcumin combined with novel therapeutic tools such as mesenchymal stem cells can offer additive neuro-generative and immunomodulatory benefits to ameliorate neurological and inflammatory diseases. Nonetheless, the beneficial effects of free curcumin decrease rapidly in aqueous conditions and its systemic bioavailability is poor. 

Micelle-encapsulated curcumin has been reported to increase the bioactivity of curcumin, enhance its solubility and promote curcumin transport towards certain targets (Alizadeh et al., 2015[2]; Yang et al., 2015[48]). Furthermore, micelles have a hydrophobic core which makes them suitable carriers for transporting bioactive materials such as curcumin into targeted cells to enhance their benefits before rapid clearance (Nusselder and Engberts, 1991[31]). Nano-micelle formulation is a potent method of preserving the antioxidant activity of curcumin, prolong its circulation time, enhance its cell penetration and protect it from systemic digestion (Kocher et al., 2015[21]; Schiborr et al., 2014[40]). 

The present *in vitro* research was designed to offer insights whether or not the combination of nanocurcuminoids and AT-MSCs can exert extra benefits for patients who undergo stem cell therapy. The effect of micelle-delivered curcumin was tested on the expansion of TCD4+CD25+FOXP3+CD127- cells (Tregs) as crucial regulating cells in repressing auto-aggressive responses in autoimmune diseases. Our findings indicate that high concentrations of micelle-curcumin (25, 59 and 100 µM) increased the Treg population by approximately 27-fold in comparison with the control groups. 

Curcumin has been established to display conflicting activity as turmeric, meaning that it decreases Treg proliferation in cancerous tissues by modifying cytotoxic T lymphocyte antigen (CTLA)-4 and shifting Tregs toward Th1 (Zhao et al., 2012[50]; Zou et al., 2018[52]). However, curcumin enhances the number of Tregs after co-culturing with normal or stem cells, presumably by stimulating TGF-b and IL2- production as vital cytokines for the proliferation of Tregs (Bhattacharyya et al., 2010[4]; Sordillo and Helson, 2015[43]) or by differentiation of naïve TCD4+ cells toward Treg cells. Altogether, curcumin exhibits an asymmetric effect on normal and cancer stem cells. A recent study has concluded that curcumin-induced immature CD4+ T cells differentiate toward CD4+CD25+CD127-Foxp3+ Tregs (Rogers et al., 2010[38]). Similarly, MSCs co-cultured with PBMCs have been shown to stimulate the generation of functional Treg cells (Khosravi et al., 2017[19]). 

Flow cytometry revealed that nanocurcuminoids remarkably inhibit MSC apoptosis at low concentrations (less than 12 µM). The MTT data also confirmed that nanocurcuminoids at low concentrations substantially stimulate MSC proliferation. This logical relation between apoptosis and proliferation after exposure to curcumin was verified in earlier investigations. Our results illustrate that low doses of micelle-encapsulated curcumin reduce AT-MSC apoptosis at least by 20-fold and enhance MSC proliferation 2.2-fold over the control cells.

In agreement with our results, a recent study has reported similar behavior of curcumin, in that high doses of curcumin (10 µM) resulted in the apoptosis of malignant cells and a low dose of curcumin (0.5 µM) elevated the proliferation rate, migration and phagocytosis of olfactory ensheathing cells (OECs) favorable for neural regeneration (Tello Velasquez et al., 2014[45]). Earlier studies have established that reduced cell viability in curcumin-treated tumor cells was accompanied by the repression of signaling proteins such as NF-ƙB and Notch-1, blocking downstream genes like vascular endothelial growth factor (VEGF), Bcl2 and matrix metalloproteinase (Marquardt et al., 2015[25]; Mou et al., 2017[30]; Pavan et al., 2016[34]). The same mechanisms may be involved in the apoptosis and proliferation of AT-MSCs after exposure to high concentrations of micelle-encapsulated curcumin. Another well-executed study has established that neural progenitor cells (NPCs) exposed to 0.5 µM of curcumin enhance neurosphere thickness, but are cytotoxic at high doses. Therefore, the careful usage of curcumin is essential in therapeutic approaches (Kim et al., 2008[20]). 

Altogether, curcumin at a concentration below 1 µM is beneficial for normal and stem cells (Cheng et al., 2013[6]; Dende et al., 2017[9]). Surprisingly, a high dose of curcumin was shown to result in apoptosis of tumor cells, but is safe for non-tumor cells. Curcumin shows different behavior in different conditions. For example, low concentrations of curcumin promote the proliferation of neural processor cells, MSCs, OECs and non-cancerous cells, whereas high doses show a cytotoxic effect on tumor cells and cancer stem cells (Tello Velasquez et al., 2014[45]). Consequently, curcumin is a double-edged sword regarding cancer and healthy stem cells. While low concentrations of curcumin are helpful for neural stem cell growth, such doses are destructive for cancer stem cells (Sordillo and Helson, 2015[43]). The justification for the conflicting functions of curcumin is that it renders a considerable effect on proliferating cells compared to quiescent cells. In addition, CXCR1 and CXCR2 are trans-membrane proteins present on cancerous cells which are potent receptors for nanocurcuminoid binding. Moreover, curcuminoid uptake is higher in malignant cell lines than in normal cells. Finally, by affecting the microenvironment of the tumor cells, modulation of tumor signaling pathways and altering the trans-differentiation of malignant cells, curcumin results in the apoptosis of cancerous cells (Sordillo and Helson, 2015[43]).

The anti-inflammatory effect of curcumin has been established in a variety of diseases, including psoriasis, colitis, and arthritis (He et al., 2015[15]). A huge number of studies have demonstrated that the anti-inflammatory effects of curcumin are exerted by reducing pro-inflammatory cytokines such as TNF, IL1, IL6 and IL17 (Ma et al., 2017[24]; Menon and Sudheer, 2007[26]). In our work, TGFB, as a key anti-inflammatory cytokine, as well as FOXP3, were upregulated at high doses of nanocurcuminoids. Similarly, the data from a recent study revealed that curcumin can enhance the expression of anti-inflammatory cytokines and FOXP3 in an animal model of inflammatory colitis (Zhao et al., 2016[51]).

The anti-inflammatory effect of curcumin can be exerted through numerous signaling pathways, including Nrf2-keap1, phosphoinositide 3-kinase (PI3K)-AKT, p38, c-Jun, PKC and AP1 (Hatamipour et al., 2018[14]). Furthermore, the anti-inflammatory effect of curcumin can be established by its effect on cyclooxygenase, inhibition of IKK activity, suppression of miRNA155, inhibition of neutrophil recruitment and overexpression of peroxisome proliferator-activated receptor-*γ *(PPAR-*γ*) activator (Ma et al., 2017[24]; Jacob et al., 2007[17]). Moreover, by blocking the ROS cascade, curcumin can inhibit downstream reactions of nuclear factor kappa-B (NF-κB) and prevent the deleterious effects of TNF mediators. Other anti-inflammatory effects of nanocurcuminoids can be instigated by shifting Th1 towards Th2 and its derivate, preparing an anti-inflammatory state (Edwards et al., 2017[10]; Rahardjo et al., 2014[36]; Toden et al., 2017[46]; Yu et al., 2018[49]). Most of these studies have proven that curcumin at higher concentrations (>20 µM) exhibit superior functionality in lowering inflammatory cytokines (Edwards et al., 2017[10]; Rahardjo et al., 2014[36]; Yu et al., 2018[49]), while we found the opposite outcome - that low doses of nanocurcuminoids are more beneficial in reducing inflammatory cytokines. 

One possible explanation for this dichotomy is that the micelle formulation of curcumin can enhance the efficacy of curcuminoids to make them more effective at low concentrations in addition to decreasing the demand for curcumin to be efficient at the same level as naked curcumin (De Jong and Borm, 2008[8]; Singh and Lillard, 2009[42]). According to the results of a recent study, curcumin at a dose of 0.5 µM for 72 h exerts stimulatory effects on NPC viability, while at 48 h, it exhibits antagonistic effects on NPC and MSC proliferation (Attari et al., 2015[3]). Therefore, if the length of nanocurcuminoid incubation is extended, it may exhibit different behavior with regard to cytokine production. 

A large number of clinical trials have linked the antioxidant effect of curcumin to the suppression of oxidant-producing enzymes such as lipoxygenase and cyclooxygenase. Methoxy and phenolic branches of curcumin are principal participators in trapping ROS (Mise Yonar et al., 2017[28]). Curcumin also protects axons from NO-mediated destruction (Tegenge et al., 2014[44]). A recent study has shown that NPCs combined with curcumin can downgrade the severity of spinal cord injury by affecting the metabolism of prostaglandin, thromboxane, NO, leukotriene, cyclooxygenase and peroxidase (Sanivarapu et al., 2016[39]).

We also evaluated the antioxidant effect of micelle-filled curcumin by measuring SOD activity and NO and MDA levels in the MSC-derived supernatant. We demonstrated that the maximum effect of nanocurcuminoids on SOD activity was 2.5 µM and that other cell concentrations were not significantly affected by nanocurcuminoids. The data also showed that the NO and MDA contents did not change significantly. Perhaps 24 h was insufficient for the optimum anti-oxidant effect of nanocurcuminoids on AT-MSCs.

The synergic effects of exosome-derived stem cells and curcumin in the restoration of an animal model of neurovascular disease were demonstrated in a recent work (Kalani et al., 2016[18]). Curcumin prevented gliosis, while MSCs restored neural lesions. Thus, MSCs conjugated with nanocurcuminoids could be advantageous for treating neurodegenerative diseases (Ormond et al., 2014[32]). Micelle-curcumin can be properly entrapped in MSCs and MSC-originated exosomes without detectable cytotoxic events (Tripodo et al., 2015[47]). This double-carrier system has an additive effect on transporting curcumin to the target location, promoting its bioavailability and protecting it from degrading agents. The ability of MSCs to correct migration into lesions is powered by insertion of the nano-micelle formulation into curcumin. Likewise, MSCs act as a drug reservoir for releasing curcumin at the demanded site. In this scenario, micelle formulation increased the accumulation of curcumin inside MSCs.

These findings recapitulate the hypothesis that nanocurcuminoids can dramatically increase the anti-apoptotic, antioxidant and immunoregulatory effects in MSCs. Taken together; the efficacy of nanocurcuminoids may differ depending on the type of stem cell, dose of the herbal component and length of incubation.

## Conclusion

Numerous studies in both basic and clinical areas have been conducted on the beneficial effects of curcumin, but little information about the efficacy of micelle-encapsulated curcumin and its optimal doses on the behavior of mesenchymal stem cells is available. Our findings demonstrate that nanocurcuminoids at very low doses assist in the proliferation of AT-MSCs and their protection from apoptosis, whereas the maximum immune-regulation effect of nanocurcuminoids on Treg expansion is exerted at high concentrations of nanocurcuminoids. The optimum doses of nanocurcuminoids for anti-oxidant effects were not conclusive. Most studies investigate the beneficial effects of MSCs at different time points and for different types of stem cells. An *in vivo* protocol must be designed to evaluate the efficacy of nanocurcuminoids in patients with/without stem cell therapy. In conclusion, the composition of micelle-encapsulated curcumin may have a beneficial synergistic effect on the treatment of patients undergoing stem cell therapy. 

## Notes

Forouzan Yousefi and Fahimeh Lavi Arab contributed equally as first authors.

## Acknowledgements

The authors wish to thank the Vice President of the Research Council of Mashhad University of Medical Sciences (MUMS) (grant number 921082). The authors declare that they have no conflict of interest.

## Supplementary Material

Supplementary data
