# Supplementary material for: Immunoregulatory, proliferative and anti-oxidant effects of nanocurcuminoids on adipose-derived mesenchymal stem cells
Source: EXCLI J. 2019 Jun 17;18:405–21. doi: 10.17179/excli2019-1366 (PMC6635727; doi:10.17179/excli2019-1366)
Supplement: Supplementary data [file EXCLI-18-405-s-001.pdf]

EXCLI Journal 2019;18:405-421 – ISSN 1611-2156
Received: April 20, 2019, accepted: May 10, 2019, published: June 17, 2019

Supplementary data to:

IMMUNOREGULATORY, PROLIFERATIVE AND ANTI-OXIDANT EFFECTS OF NANOCURCUMINOIDS ON ADIPOSE-DERIVED MESENCHYMAL STEM CELLS

Forouzan Yousefi[a#], Fahimeh Lavi Arab[a#], Mahmoud Reza Jaafari[b,c], Maryam Rastin[d], Nafiseh Tabasi[d], Mahdi Hatamipour[e], Karim Nikkhah[f], Mahmoud Mahmoudi[a,g*]

[a] Immunology Research Center, Mashhad University of Medical Sciences, Mashhad, Iran
[b] Biotechnology Research Center, Pharmaceutical Technology Institute, Mashhad University of Medical Sciences, Mashhad, Iran
[c] Department of Pharmaceutical Nanotechnology, School of Pharmacy, Mashhad University of Medical Sciences, Mashhad, Iran
[d] Immunology Research Center, BuAli Research Institute, Faculty of Medicine, Mashhad University of Medical Sciences, Mashhad, Iran
[e] Nanotechnology Research Center, Mashhad University of Medical Sciences, Mashhad, Iran
[f] Department of Neurology, Faculty of Medicine, Mashhad University of Medical Sciences, Mashhad, Iran
[g] Department of Immunology, Faculty of Medicine, Mashhad University of Medical Sciences, Mashhad, Iran.

[#] Co-first authors: F.Y. and F.L. contributed equally to this research.
* Corresponding author: Mahmoud Mahmoudi, Department of Immunology, Faculty of Medicine, Mashhad University of Medical Sciences, Mashhad, Iran. Tel: +98 9151156304, Fax: +98 5138022229, E-mail: MahmoudiM@mums.ac.ir

http://dx.doi.org/10.17179/excli2019-1366

This is an Open Access article distributed under the terms of the Creative Commons Attribution License (http://creativecommons.org/licenses/by/4.0/).

EXCLI Journal 2019;18:405-421 – ISSN 1611-2156
Received: April 20, 2019, accepted: May 10, 2019, published: June 17, 2019

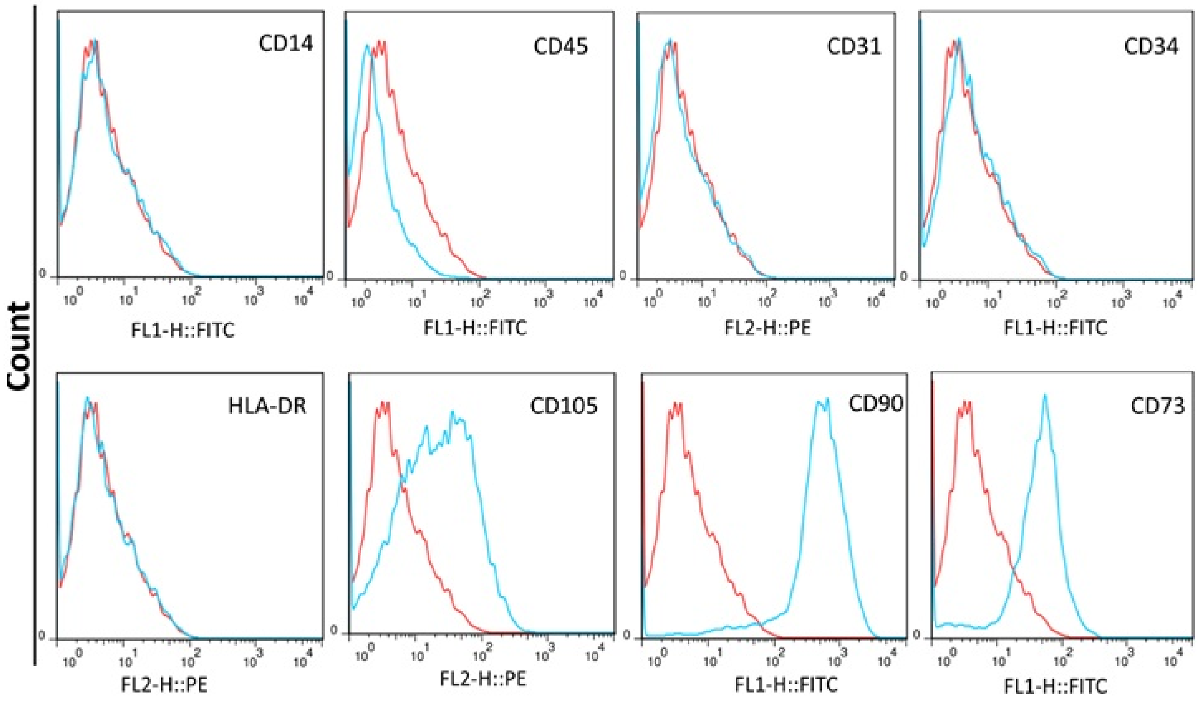

Supplementary Figure 1: Flow cytometry analysis of superficial markers of AT-MSCs based on comparison with negative isotype control on 10,000 evens. Histogram plots show that AT-MSCs are positive for expression of CD73, CD105 and CD90, but negative for CD14, CD34, CD31, CD45 and HLA-DR.

EXCLI Journal 2019;18:405-421 – ISSN 1611-2156
Received: April 20, 2019, accepted: May 10, 2019, published: June 17, 2019

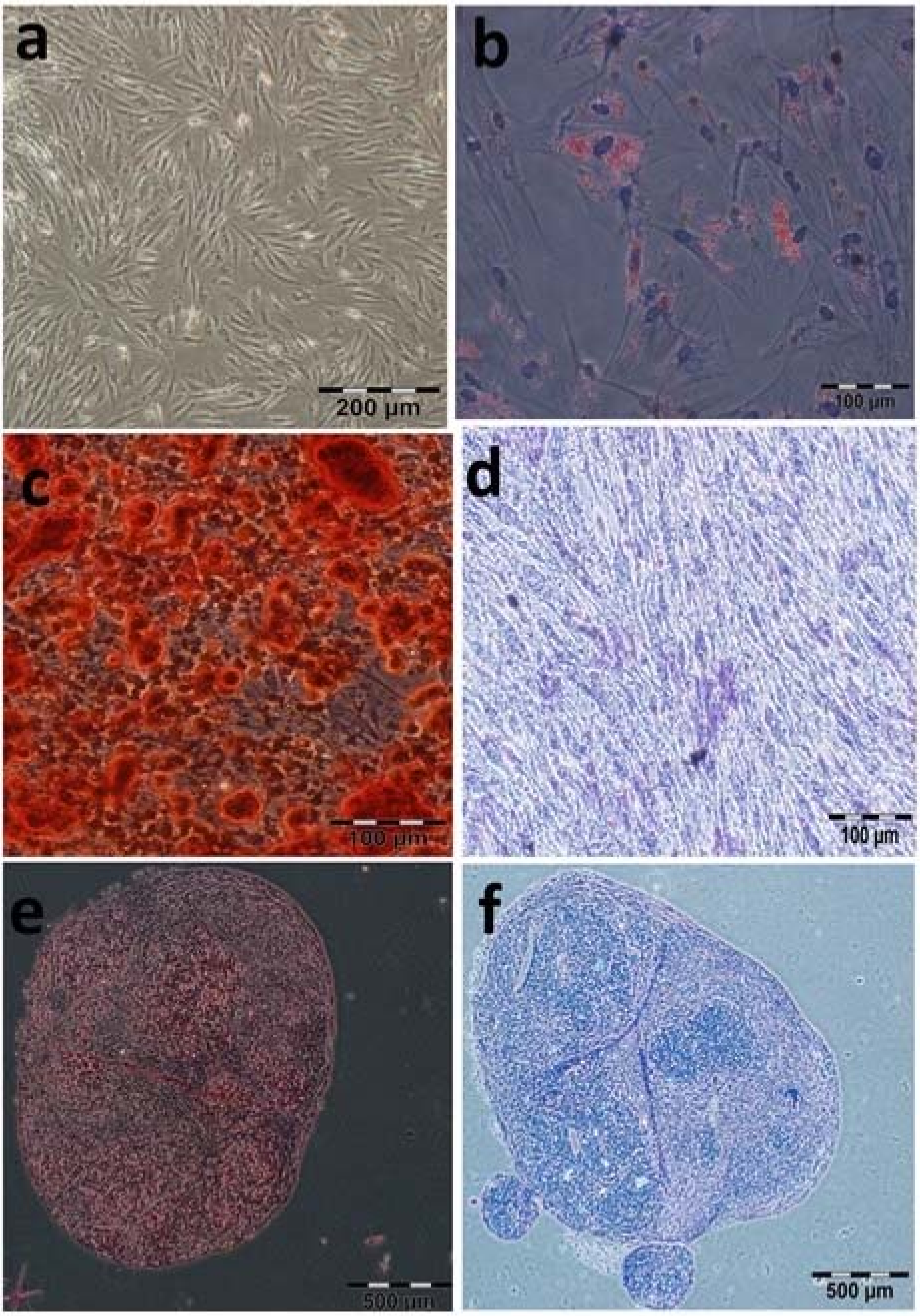

Supplementary Figure 2: Tri-lineage capacity of AT-MSCs for differentiation into adipocytes, osteocytes and chondrocytes established by specific-cell staining after 21 days in a specialized medium: (**a**) fibroblast-like shape of AT-MSCs before differentiation; (**b**) and after differentiation of AT-MSCs toward adipocytes with lipid vacuoles detected by Oil Red O staining; (**c**) mineralization potential of AT-MSCs with appearance of calcium deposits following alizarin red staining; (**d**) and after alkaline phosphatase staining; (**e**) GAGs and sulfated proteoglycans from chondroitin cryosections after staining with hematoxylin-eosin; (**f**) and after toluidine blue.
